# Prevalence and subtype distribution of *Blastocystis* infections among community participants in Thailand: a systematic review and meta-analysis

**DOI:** 10.1051/parasite/2025042

**Published:** 2025-08-19

**Authors:** Manas Kotepui, Supaluk Popruk, Kwuntida Uthaisar Kotepui, Frederick Ramirez Masangkay, Kinley Wangdi, Aongart Mahittikorn, Christen Rune Stensvold

**Affiliations:** 1 Medical Technology Program, Faculty of Science, Nakhon Phanom University Nakhon Phanom 48000 Thailand; 2 Department of Protozoology, Faculty of Tropical Medicine, Mahidol University Bangkok 10400 Thailand; 3 Department of Medical Technology, Faculty of Pharmacy, University of Santo Tomas Manila 1008 Philippines; 4 HEAL Global Research Centre, Health Research Institute, Faculty of Health Bruce ACT 2617 Australia; 5 National Centre for Epidemiology and Population Health, Australian National University Acton ACT 2601 Australia; 6 Laboratory of Parasitology, Department of Bacteria, Parasites and Fungi, Statens Serum Institut Artillerivej 5 DK–2300 Copenhagen S Denmark

**Keywords:** *Blastocystis*, *Blastocystis hominis*, Subtype, Thailand, Prevalence

## Abstract

A comprehensive understanding of the prevalence and subtype distribution of *Blastocystis* infections among community participants in Thailand is essential to inform targeted public health interventions. This systematic review and meta-analysis aimed to estimate the overall prevalence of *Blastocystis* infections and to determine the distribution of subtypes among community participants in Thailand. Relevant studies on *Blastocystis* infections in community participants in Thailand were searched in PubMed, Embase, Scopus, Ovid, ProQuest, and the Thai-Journal Citation Index. The methodological quality of the included studies was assessed using the Joanna Briggs Institute critical appraisal tools. Prevalence estimates and subtype distributions were calculated using random-effects models. A total of 947 articles were identified, with 60 studies included in the systematic review and meta-analysis. The meta-analysis led to an estimated overall prevalence of *Blastocystis* infections in community participants in Thailand at 8.34% (95% CI: 5.48%–12.51%; *I*^2^: 98.2%; number of studies: 60; number of participants: 33,101). Meta-regression analysis showed no significant temporal trends in infection prevalence. The highest prevalence rates were observed in Eastern Thailand (13.54%) and Western Thailand (10.09%). Subtype analysis identified ST3 and ST1 as the most common subtypes, accounting for 50.05% and 23.50% of positive samples, respectively. The highest prevalence was reported in military personnel (29.87%), followed by orphans (29.01%). Improved use of molecular and culture-based diagnostic methods is recommended to enhance detection accuracy. Public health interventions should prioritize high-risk groups, such as military personnel and orphans, and address regional disparities to reduce the burden of *Blastocystis* infections.

## Abstract (fr)

Une compréhension approfondie de la prévalence des infections à *Blastocystis* et de la distribution des sous-types chez les participants communautaires en Thaïlande est essentielle pour éclairer les interventions de santé publique ciblées. Cette revue systématique et méta-analyse visait à estimer la prévalence globale des infections à *Blastocystis* et à déterminer la distribution des sous-types parmi les participants communautaires en Thaïlande. Des études pertinentes sur les infections à *Blastocystis* chez les participants communautaires en Thaïlande ont été consultées dans PubMed, Embase, Scopus, Ovid, ProQuest et le Thai-Journal Citation Index. La qualité méthodologique des études incluses a été évaluée à l’aide des outils d’évaluation critique du Joanna Briggs Institute. Les estimations de prévalence et les distributions des sous-types ont été calculées à l’aide de modèles à effets aléatoires. Au total, 947 articles ont été identifiés, dont 60 études incluses dans la revue systématique et la méta-analyse. La méta-analyse a estimé la prévalence globale des infections à *Blastocystis* chez les participants communautaires en Thaïlande à 8,34 % (IC à 95 % : 5,48 %–12,51 % ; *I*^2^ : 98,2 %; nombre d’études : 60 ; nombre de participants : 33 101). L’analyse de méta-régression n’a montré aucune tendance temporelle significative dans la prévalence de l’infection. Les taux de prévalence les plus élevés ont été observés dans l’est (13,54 %) et dans l’ouest de la Thaïlande (10,09 %). L’analyse des sous-types a identifié ST3 et ST1 comme les sous-types les plus courants, représentant respectivement 50,05 % et 23,50 % des échantillons positifs. La prévalence la plus élevée a été signalée chez le personnel militaire (29,87 %), suivi des orphelins (29,01 %). Une meilleure utilisation des méthodes de diagnostic moléculaire et basées sur la culture est recommandée pour améliorer la précision de la détection. Les interventions de santé publique devraient donner la priorité aux groupes à haut risque, tels que le personnel militaire et les orphelins, et s’attaquer aux disparités régionales pour réduire le fardeau des infections à *Blastocystis*.

## Introduction

*Blastocystis* species are protozoan parasites transmitted via the fecal-oral route and commonly colonize the gastrointestinal tracts of humans and animals [28, 67, 79]. The parasite exhibits six distinct morphological forms: avacuolar, vacuolar, multivacuolar, granular, amoeboid, and cystic. The cyst is the infective stage, while the amoeboid form may contribute more directly to symptom development [35]. Clinical manifestations of *Blastocystis* infections can be asymptomatic or symptomatic, including nausea, abdominal pain, diarrhea, bloating, and irritable bowel syndrome (IBS) [19, 28, 63]. Although sometimes regarded as commensals, a few researchers consider *Blastocystis* spp. to be neglected pathogens [5].

*Blastocystis* demonstrates substantial genetic diversity, with over 44 subtypes (STs) identified through sequence analysis of the small subunit ribosomal RNA (SSU rRNA) gene [50]. Globally, most human infections are caused by subtypes ST1 to ST4, with ST3 being the most prevalent [50, 79]. Among these, ST1 is often associated with asymptomatic carriage, whereas ST4 has been linked to symptomatic cases [50]. Certain populations face a higher risk, particularly immunocompromised individuals, such as those with HIV/AIDS or cancer [29, 30, 75]. In addition, close contact with animals, poor sanitation, and unsafe water sources further elevate the risk of infection [1, 3, 28, 43].

In Thailand, the prevalence and distribution of *Blastocystis* subtypes vary across population groups and regions. For example, one cross-sectional study in Thai children reported a 13.6% prevalence, with ST3 as the predominant subtype (80.0%), followed by ST2 (12.5%) and ST1 (8.0%) [48]. Another study among children found a lower prevalence of 3.35%, though ST3 remained the dominant subtype (64.7%) [2]. In contrast, orphans in institutional settings showed a much higher infection rate of 51.2%, with ST3 accounting for 79.69% of cases [46]. These findings highlight significant variation in *Blastocystis* prevalence based on population characteristics.

Although many studies have examined regional prevalence, no systematic summary of *Blastocystis* infections and subtypes in Thai community populations has been conducted. This systematic review and meta-analysis aimed to estimate the pooled prevalence of *Blastocystis* infections and to characterize the distribution of subtypes among community participants in Thailand. The results may inform the development of targeted public health interventions and control strategies aimed at reducing the burden of *Blastocystis* infections in the general Thai population.

## Methods

### Protocol registrations

The study protocol was registered at PROSPERO (CRD42024621794). The systematic review followed the Preferred Reporting Items for Systematic Reviews and Meta-Analyses (PRISMA) guidelines [41].

### Systematic review questions

Research questions for this systematic review and meta-analysis were formulated using the population, exposure, comparator, and outcomes (PECO) framework [40]. The population (P) was community participants (Thai participants who did not seek medical treatment at hospitals or health centers) in Thailand; exposure (E) was the detection of *Blastocystis* infections by any method; comparator (C) was not applicable; and outcome (O) was the prevalence/proportion estimates of *Blastocystis* infections among community participants in Thailand.

### Eligibility criteria

The inclusion criteria focused on original research articles investigating *Blastocystis* infections using fecal samples. The participants were Thai people living in Thailand. Studies were excluded if they were unrelated to *Blastocystis*, conducted outside Thailand, or involved non-Thai participants. Only community participants, such as villagers, school and preschool children, military personnel, pig handlers, children and caregivers, food handlers, gardeners, monks or nuns, laborers, and participants in annual check-up programs, were considered. Classification of participants was based on the context described in the original studies. Population groups were defined as follows: “school children” referred to participants enrolled in primary or secondary school, typically aged 6–17 years; “preschool children” were defined as children under the age of 6 not yet enrolled in formal primary education; “orphans” referred to children living in institutional care settings, which may include a wide age range, generally from infancy to adolescence. Orphans may overlap demographically with school or preschool-aged children; however, they were treated as a distinct group due to their unique living conditions (e.g., institutional care, shared facilities) and increased vulnerability to infection risks, such as close-contact transmission and poor sanitation. Studies on non-human samples, hospitalized patients, *in vitro* or *in vivo* experiments, and test performance assessments were excluded. Additionally, conference abstracts, review articles, systematic reviews, and case series were not included. Articles published in English and Thai were considered, with no restrictions on publication year.

### Search strategy

The search strategy involved a systematic approach using multiple databases to identify relevant studies on *Blastocystis* infections in community participants in Thailand. The search terms included variations of *Blastocystis* (e.g., *Blastocystis hominis*, *Blastocysti*, *blastocystina*) combined with geographical terms (Thailand or Siam). Boolean operators (AND, OR) were used to refine searches to studies that included both the pathogen and the location. The literature search was conducted across six major databases: PubMed, Embase, Scopus, Ovid, ProQuest, and the Thai-Journal Citation Index (TCI) (Table S1). The reference lists of included studies were also reviewed to identify relevant articles that met the eligibility criteria.

### Study selection and data extraction

After retrieving articles from databases, they were imported into EndNote version 21.0 (Philadelphia, PA, USA) for study selection. First, articles were screened for relevance based on their titles and abstracts, and non-relevant articles were excluded. Second, the remaining articles were assessed in full text according to the inclusion and exclusion criteria. Articles that did not meet the criteria were excluded, with specific reasons documented. Eligible articles were then used for data extraction, which was performed using Microsoft Excel 2021 (Microsoft Corporation, Redmond, WA, USA). The extracted data included the first author’s surname, publication year, study design, study location, region in Thailand, year of study conduct, types and number of participants, age range, percentage of male participants, number of *Blastocystis* infections, identified *Blastocystis* subtypes, and the method used for *Blastocystis* detection. Study selection and data extraction were independently performed by two authors (MK, AM). Any disagreements were resolved through discussion to reach a consensus.

### Risk of bias assessment

The methodological quality of the included studies was assessed using the Joanna Briggs Institute (JBI) critical appraisal tools [39]. The risk of bias assessment was independently conducted by two authors (MK, AM), and any disagreements were resolved through discussion to reach a consensus.

### Data synthesis and statistical analysis

The prevalence and proportion estimates were pooled using random-effects models [13]. The primary outcome was the prevalence of *Blastocystis* infections among community participants in Thailand. The secondary outcome was the proportion of *Blastocystis* subtypes among community participants in Thailand. Heterogeneity was assessed using the *I*^2^ statistic, with values of 25%, 50%, and 75% indicating low, moderate, and high heterogeneity, respectively [16]. The potential sources of heterogeneity were investigated using meta-regression, with publication year, study design, region in Thailand, age range, percentage of male participants, and the method used for *Blastocystis* detection as covariates. Subgroup analysis was conducted to assess differences in pooled effect estimates based on publication year, study design, region in Thailand, participant types, age range, and the method used for *Blastocystis* detection. A funnel plot and Egger’s regression test were used to identify publication bias in the meta-analysis if at least 10 studies were included [17]. Statistical analysis was performed using RStudio (Version: 2024.04.2+764) [73].

## Results

### Search results

**Figure 1:**
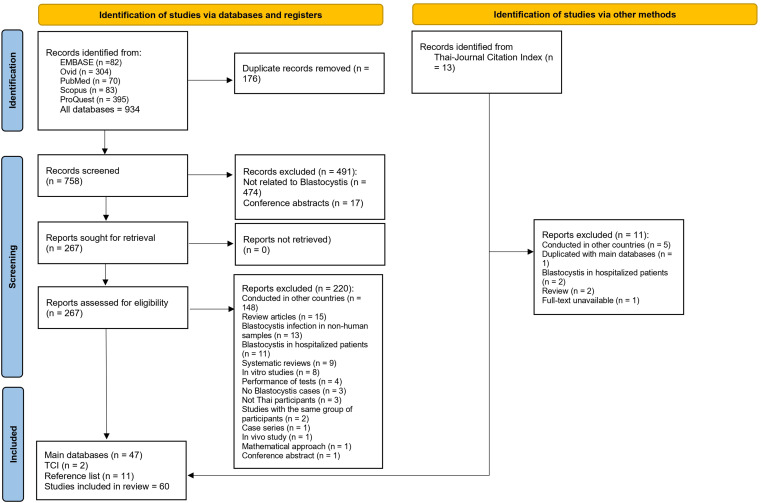
PRISMA flow diagram.

Initially, 934 records were identified from six databases (EMBASE, MEDLINE, Ovid, PubMed, Scopus, and ProQuest), along with 13 additional records from the Thai-Journal Citation Index (TCI), totaling 947 records. Finally, 60 studies were included in the systematic review and meta-analysis [2, 4, 6–10, 12, 18, 20–27, 31–34, 37, 38, 42, 44–49, 51–55, 57, 59–62, 64–66, 68, 69, 71, 72, 74, 76–78, 80–88], comprising 47 from the main databases [2, 4, 6, 7, 9, 18, 20, 22, 24–27, 31–34, 37, 42, 47–49, 51–55, 59–62, 64, 66, 68, 69, 71, 72, 74, 76–78, 80–82, 84–86, 88], 2 from TCI [21, 46], and 11 from reference lists [8, 10, 12, 23, 38, 44, 45, 57, 65, 83, 87] (Fig. 1).

### Summary of study designs, populations, and diagnostic methods

The summary characteristics of the 60 included studies are shown in Table 1. A high proportion of studies (43%) were published between 2010 and 2019, and most (95%) employed a cross-sectional design. Geographically, most studies were conducted in Central Thailand (33%), followed by Eastern (20%), Northern (13%), Western (10%), Northeastern (10%), and Southern Thailand (7%), with a few spanning multiple regions (7%). Regarding patient demographics, villagers (28%) and schoolchildren (22%) were the most studied groups, while other populations included military personnel (8%), hill-tribe children (5%), orphans (5%), and preschool children (3%). Several studies also involved specific occupational groups such as pig handlers, food handlers, gardeners, and monks. For *Blastocystis* detection, the concentration method (alone or combined with a direct smear) was most used (42%), followed by molecular methods (32%), culture-based approaches (17%), and direct smear-only (10%). Details of each study are demonstrated in Table S2.

**Table 1:** Summary characteristics of included studies.

Characteristics	*n* (60 studies)	%
Publication year		
2010–2019	26	43.0
2000–2009	19	32.0
2020–2024	14	23.0
Before 2000	1	2.00
Study designs		
Cross-sectional study	57	95.0
Cohort study	3	5.00
Study regions		
Central Thailand	20	33.0
Eastern Thailand	12	20.0
Northern Thailand	8	13.0
Western Thailand	6	10.0
Northeastern Thailand	6	10.0
Southern Thailand	4	7.00
More than two regions	4	7.00
Types of participants		
Villagers	17	28.0
School children	13	22.0
Military personnel	5	8.00
Hill-tribe children	3	5.00
Orphans	3	5.00
Orphans, childcare workers	2	3.00
Preschool children	2	3.00
School children, villagers	2	3.00
Pig handlers, villagers near pig farms	2	3.00
Mentally disabled people	2	3.00
School children, preschool children	2	3.00
Children and caregivers	1	1.50
Food handlers	1	1.50
Gardeners	1	1.50
Monks or nuns, villagers	1	1.50
Participants at a Home for Girls	1	1.50
Thai laborers	1	1.50
Participants who enrolled in annual check-up programs	1	1.50
Diagnostic method for *Blastocystis*		
Concentration method (alone or with direct smear)	25	42.0
Molecular method (alone or with other methods)	19	32.0
Culture (with non-molecular methods)	10	17.0
Direct smear (only)	6	10.0

### Risk of bias across included studies

The risk of bias assessment for analytical cross-sectional studies revealed that most studies included in the analysis met key quality criteria (Table S3). Nearly all studies had clearly defined inclusion criteria, adequately described study subjects and settings, and used valid and reliable methods for measuring exposure and outcomes. However, a notable limitation across several studies was the lack of identification and control of confounding factors [2, 9, 21–23, 26, 27, 34, 42, 46, 47, 51, 52, 61, 62, 64–66, 68, 71, 80, 81]. Furthermore, certain studies [45, 61, 77, 85, 86, 88] had unclear reporting on statistical analysis or incomplete follow-up strategies.

The risk of bias assessment for the three cohort studies showed overall methodological strength, with all studies meeting key criteria such as comparable group recruitment, valid exposure and outcome measurements, identification of confounding factors, and appropriate statistical analysis. However, potential bias exists due to incomplete follow-up reporting, as two studies [7, 84] did not fully address loss to follow-up, and one study [49] had unclear follow-up completeness. Additionally, two studies [7, 49] did not clearly outline strategies for handling incomplete follow-up.

### Prevalence of *Blastocystis* infections in Thailand

**Figure 2:**
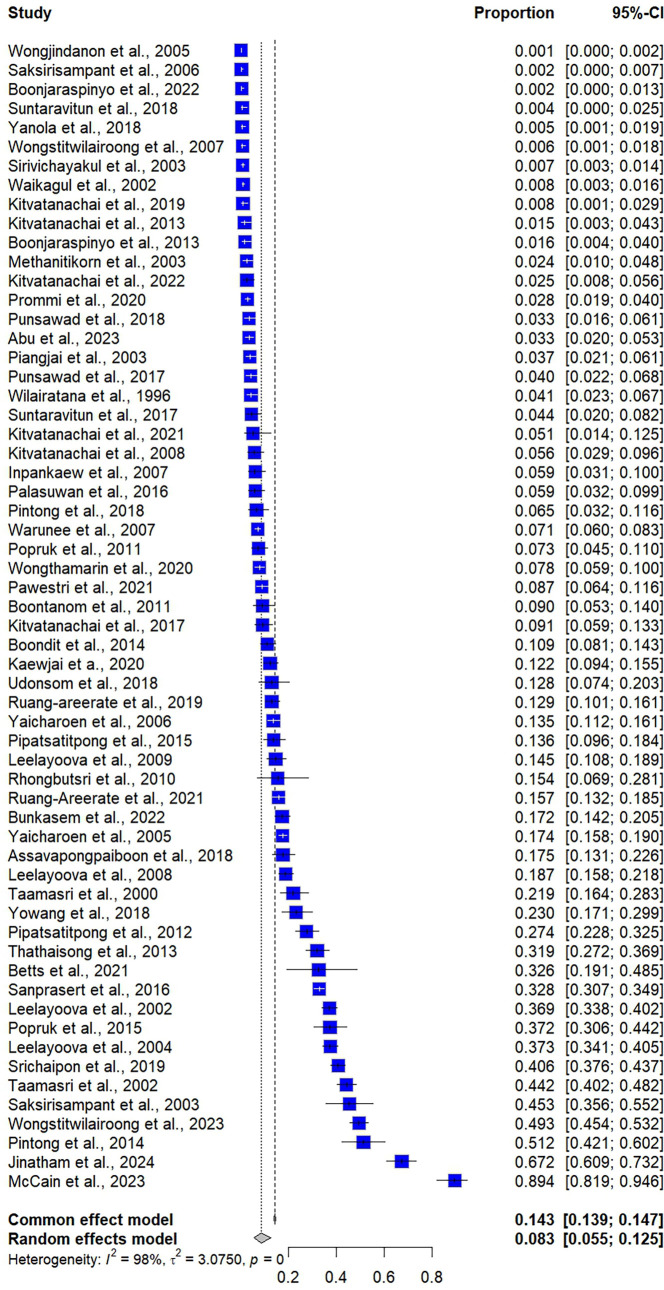
Forest plot presenting the prevalence estimate of *Blastocystis* infections in community participants in Thailand (proportion × 100 = prevalence estimate). A blue square represents an individual study, and the square’s size reflects the study’s weight in the meta-analysis. The horizontal lines extending from the squares are the 95% confidence interval (CI) for each study’s prevalence estimate. The vertical dashed line is the overall effect estimate.

**Figure 3:**
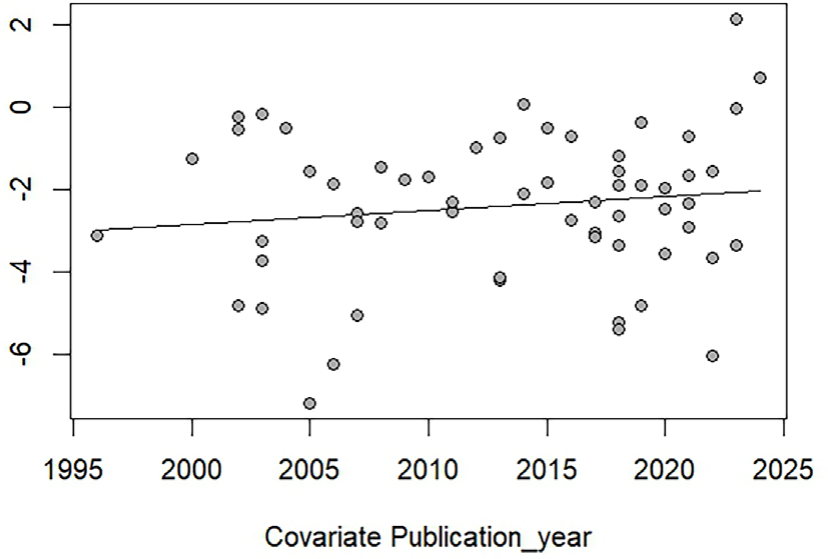
Bubble plot presenting the distribution of the prevalence estimate of *Blastocystis* infections over time (log-prevalence estimates between 1995 and 2024). Each bubble represents an individual study, with the *X*-axis being the publication year and the *Y*-axis being the log-prevalence estimate. The line of best fit shows the overall trend in the data.

The meta-analysis using random-effect models revealed that the overall prevalence estimate of *Blastocystis* infections in community participants in Thailand was 8.34% (95% CI: 5.48%–12.51%, *I*^2^: 98.2%, number of studies: 60, number of participants: 33,101; Fig. 2). The meta-regression analysis revealed that the high heterogeneity of the prevalence estimates may be influenced by the difference in regions of Thailand (*p* = 0.0053) and varying methods for *Blastocystis* detection (*p* < 0.0001) (Table S4). Subgroup analyses revealed that regions of Thailand (*p* < 0.0001), types of participants (*p* < 0.0001), and methods for *Blastocystis* detection (*p* < 0.0001) showed different prevalence estimates (Table 2). There were no significant alterations in the trends of *Blastocystis* infections over time between the years between 1995 and 2024 (Fig. 3).

**Table 2:** Subgroup analysis.

Pooled proportion	Test for subgroup difference	Prevalence estimate (%) (95% CI)	*I*^2^ (%)	Number of studies
Overall		8.34 (5.48–12.51)	98.2	60
Publication year	0.0815			
Before 2000		4.14 (2.51–6.76)	N/A	1
2000–2009		5.91 (2.47–13.47)	98.6	19
2010–2019		19.79 (18.98–20.63)	96.9	26
2020–2024		16.16 (15.28–17.09)	98.7	14
Study design	0.1929			
Cross-sectional study		8.10 (5.20–12.41)	98.2	57
Cohort study		13.57 (7.05–24.55)	97.3	3
Regions of Thailand	<0.0001			
Eastern Thailand		13.54 (7.41–23.44)	97.9	12
Western Thailand		10.09 (2.83–30.17)	98.7	6
Central Thailand		8.85 (4.89–15.48)	96.8	20
Northern Thailand		7.11 (1.49–27.91)	98.2	8
Northeastern Thailand		4.73 (1.51–13.84)	88.5	6
Southern Thailand		2.79 (1.96–3.97)	39.2	4
Northern, Eastern Thailand		67.23 (61.01–72.89)	N/A	1
Central, Northeastern, Northern, Eastern, Western, Southern Thailand		32.79 (30.72–34.93)	N/A	1
Central, Northern, Northeastern, Western Thailand		40.59 (37.62–43.62)	N/A	1
Central, Northeastern Thailand		0.07 (0.02–0.23)	N/A	1
Age groups				
Children	0.7490	6.05 (2.35–14.71)	98.3	19
Adults		11.68 (4.07–29.20)	98.4	11
All age groups		8.23 (4.57–14.38)	97.7	19
Not specified		10.45 (5.03–20.44)	97.9	11
Types of participants	<0.0001			
School children		7.50 (2.63–19.59)	98.6	13
Villagers		6.31 (2.89–13.21)	97.7	17
Military personnel		29.87 (20.61–41.13)	95.6	5
Orphans		29.01 (9.77–60.67)	97.7	3
Hill-tribe children		1.87 (0.79–4.37)	71.8	3
Orphans, childcare workers		17.75 (8.98–32.07)	96.9	2
Preschool children		2.48 (0.36–15.32)	94.7	2
School children, villagers		1.20 (0.02–39.99)	98.9	2
Mentally disabled people		1.41 (0.86–2.28)	97.3	2
School children, preschool children		40.66 (29.80–52.51)	98.2	2
Pig handlers, villagers near pig farms		9.21 (5.69–14.57)	67.4	2
Monks or nuns, villagers		5.88 (3.37–10.07)	N/A	1
Gardeners		9.09 (6.12–13.31)	N/A	1
Food handlers		5.06 (1.91–12.73)	N/A	1
Children and caregivers		13.57 (9.90–18.31)	N/A	1
Participants at a Home for Girls		31.89 (27.34–36.82)	N/A	1
Thai laborers		4.14 (2.51–6.76)	N/A	1
Participants who enrolled in annual check-up programs		17.40 (15.88–19.03)	N/A	1
Methods of *Blastocystis* detection	< 0.0001			
Molecular method (alone or with other methods)		21.42 (13.31–32.61)	97.9	19
Concentration method (alone or with direct smear)		3.43 (1.85–6.29)	98.1	25
Culture (with non-molecular methods)		22.11 (14.43–32.34)	98.5	10
Direct smear (only)		1.79 (0.45–6.86)	93.0	6

N/A, not assessed.

**Figure 4:**
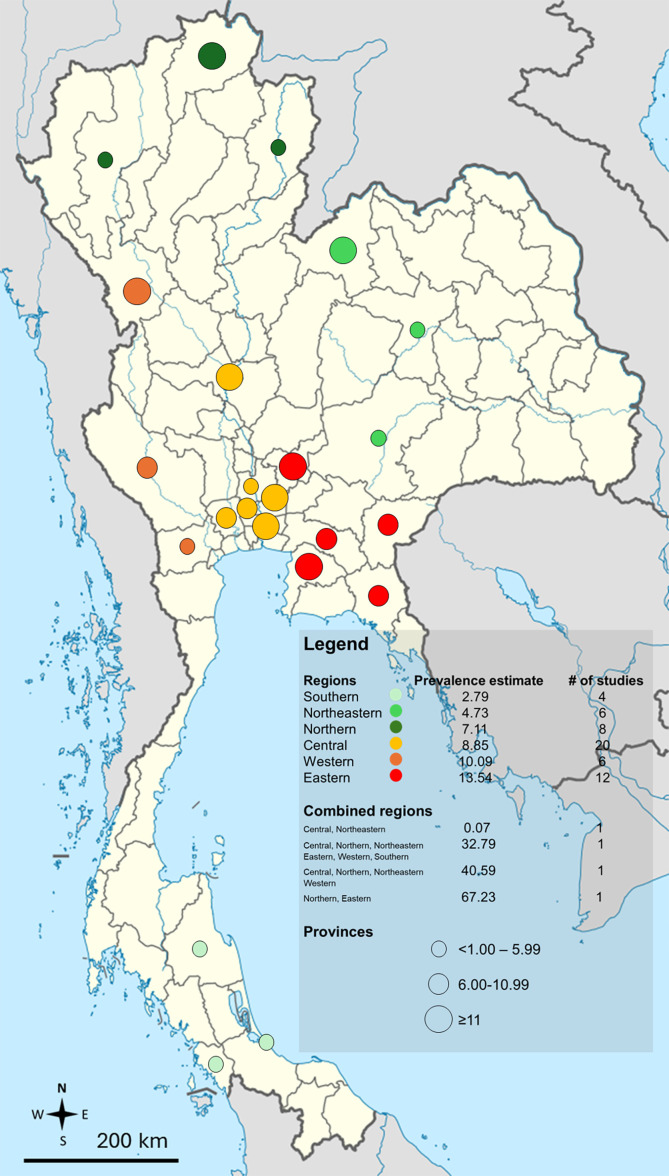
Geographic distribution of the proportion of *Blastocystis* in community participants in Thailand.

The subgroup analysis showed a higher prevalence estimate of *Blastocystis* infections in community participants in Thailand among cohort studies that assessed prevalence at baseline (13.57%, 3 studies) compared to cross-sectional studies (8.10%, 57 studies). Regarding regional differences, the highest prevalence estimate was observed in Eastern Thailand (13.54%, 12 studies), followed by Western Thailand (10.09%, 6 studies), Central Thailand (8.85%, 20 studies), Northern Thailand (7.11%, 8 studies), Northeastern Thailand (4.73%, 6 studies), and Southern Thailand (2.79%, 4 studies). Regarding age group differences, studies that enrolled adults reported a higher prevalence estimate (11.68%) than those involving children (6.05%). The geographic distribution of the *Blastocystis* infections in community participants in Thailand is demonstrated in Figure 4.

The prevalence estimates of *Blastocystis* infections in community participants in Thailand varied significantly among different types of participants (*p* < 0.0001, Table 2). Based on the meta-analysis, which included three or more studies, the highest prevalence estimate was observed in military personnel (29.87%, 5 studies), followed by orphans (29.01%, 3 studies), school children (7.50%, 13 studies), and villagers (6.31%, 17 studies). The method used for *Blastocystis* detection also significantly influenced prevalence estimates (*p* < 0.0001). The highest prevalence estimate was reported in studies using culture with non-molecular methods (22.11%, 10 studies), followed by molecular methods used alone or combined with other methods (21.42%, 19 studies), concentration methods alone or combined with the direct smear method (3.43%, 25 studies), and the direct smear only (1.79%, 6 studies).

The distribution of *Blastocystis* subtypes in community participants in Thailand based on 820 positive samples is summarized in Table 3. ST3 was the most prevalent, with a proportion estimate of 50.05% and a crude proportion of 47.68%, followed by ST1 at 23.50% and 27.44%, for proportion estimate and crude proportion, respectively. ST2 was less common, with a proportion estimate of 6.10% and a crude proportion of 8.66%. Other subtypes, including ST4, ST6, ST7, and ST10, had minimal prevalence, with each comprising less than 1.00% of cases. These findings indicate that ST3 and ST1 are the dominant *Blastocystis* subtypes in Thailand, whereas other subtypes are rarely detected.

**Table 3:** Distribution of *Blastocystis* subtypes in community participants in Thailand (total positive samples = 820).

Subtypes	Proportion estimate (%)	95% CI (%)	*I*^*2*^ (%)	Number of studies	Observed (*n*)	Crude proportion (%, 820 samples)
Subtype 1	23.50	14.84–35.12	88.0	15	225	27.44
Subtype 2	6.10	2.70–13.21	68.4	13	71	8.66
Subtype 3	50.05	32.68–67.42	82.5	15	391	47.68
Subtype 4	0.41	0.13–1.27	0.0	14	3	0.37
Subtype 5	*	*	*	*	7	0.85
Subtype 6	0.52	0.11–2.30	0.0	14	5	0.61
Subtype 7	0.28	0.02–4.43	0.0	14	55	6.71
Subtype 10	0.01	0.00–72.79	0.0	14	4	0.49
Subtype 23	*	*	*	*	9	1.10
Subtype 26	0.12	0.02–0.86	0.0	15	1	0.12
Mixed Subtype	*	*	*	*	21	2.56
Unknown subtype	0.43	0.04–4.94	0.0	15	27	3.29

N/A, not assessed; *, unable to calculate the proportion estimate

### Sensitivity analysis

**Figure S1.**
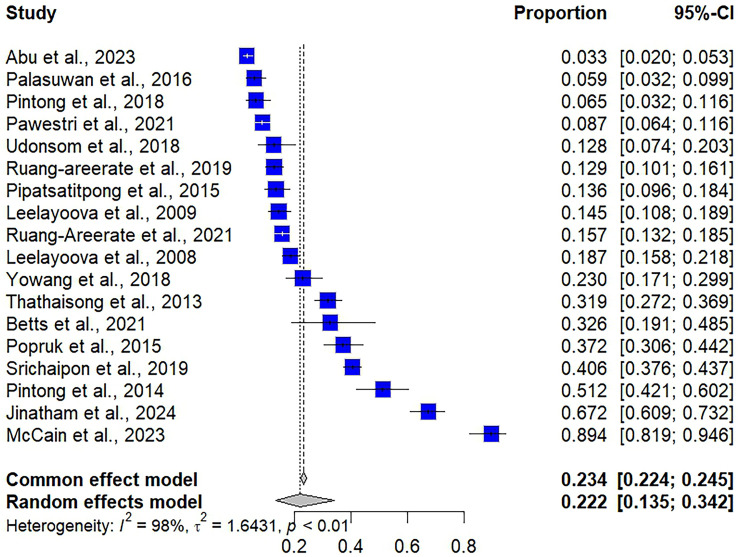
Prevalence by molecular method.

**Figure S2.**
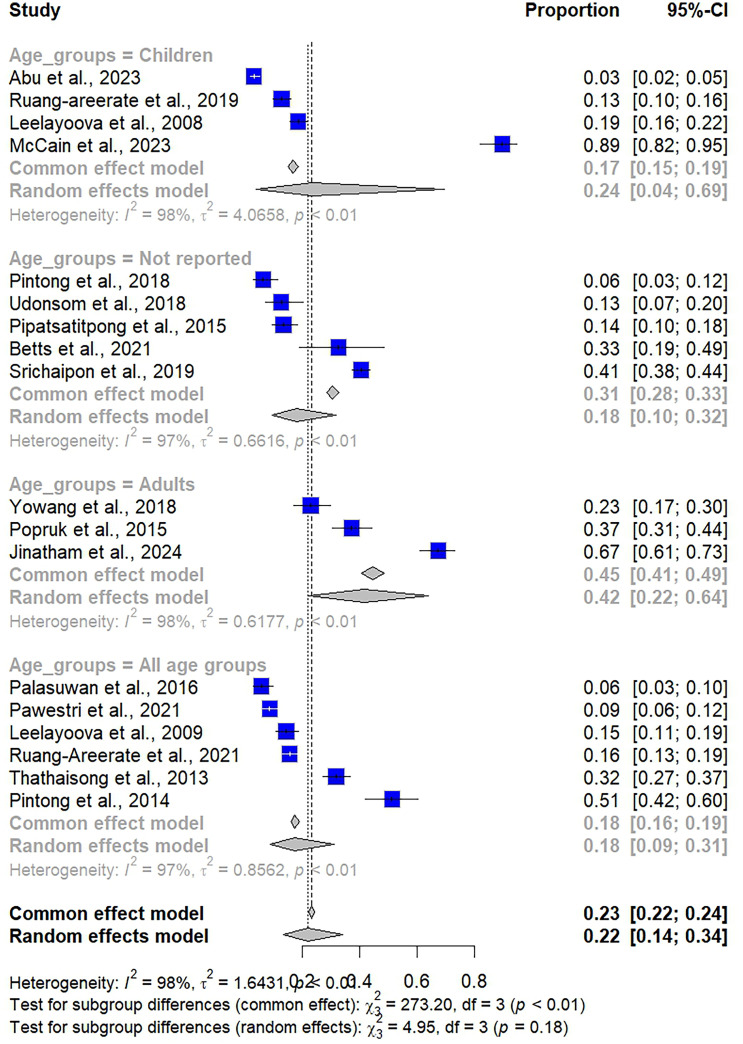
Prevalence by molecular method: age groups.

The fixed effect model demonstrated that the prevalence estimates of *Blastocystis* infections among community participants in Thailand were 14.28% (95% CI: 13.91%–14.66%, Fig. 2). Among cross-sectional studies that used molecular methods alone or combined with other methods, the estimated prevalence of *Blastocystis* infections among community participants in Thailand was 22.19% based on a random-effects model (95% CI: 13.54%–34.18%, *I*^2^: 97.9%, 18 studies, Supplementary Fig. S1). Further subgroup analysis revealed that the prevalence of *Blastocystis* infections in children was 23.80% (95% CI: 4.10%–69.50%, *I*^2^: 98.5%, 4 studies, Fig. S2), while the prevalence in adults was 41.73% (95% CI: 22.44%–63.93%, *I*^2^: 97.5%, 3 studies).

### Publication bias

**Figure 5:**
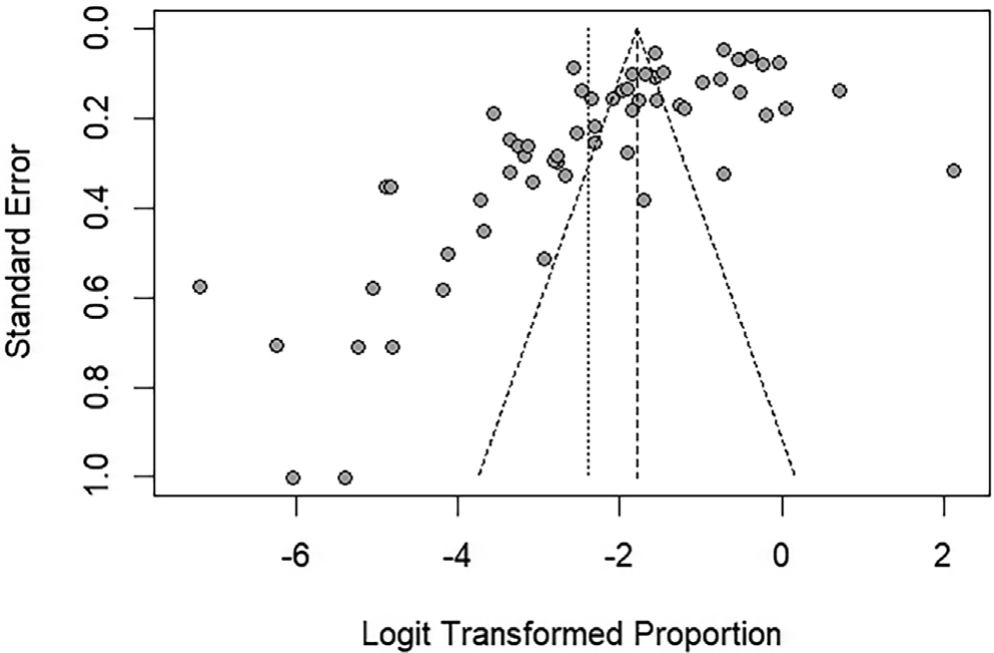
Funnel plot assessing publication bias in the meta-analysis. The plot displays the standard error versus the logit-transformed proportion of *Blastocystis* infections. The gray dots are the prevalence estimates from each study. The asymmetrical distribution of points around the central line suggests potential bias in the included studies.

The funnel plot assessing publication bias in the meta-analysis revealed the asymmetrical distribution of points around the central line, suggesting potential bias in the included studies (Fig. 5). Egger’s regression test of funnel plot asymmetry revealed significant funnel plot asymmetry (*p* < 0.001), indicating publication bias in the meta-analysis of the prevalence estimate.

**Figure S3.**
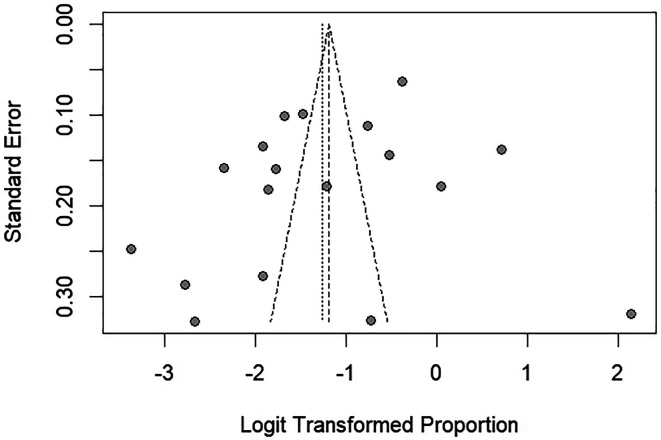
Funnel plot, ST.

**Figure S4.**
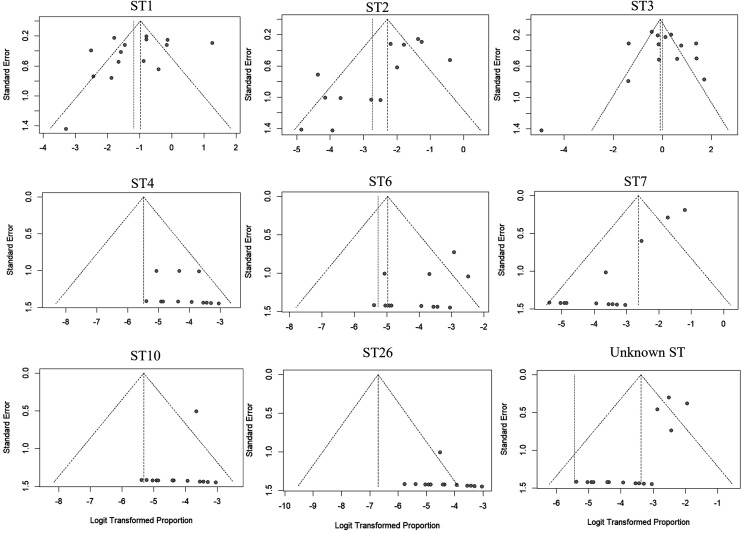
Funnel plot, molecular.

For the meta-analysis of the proportion estimates of *Blastocystis* subtypes, the funnel plot demonstrated an asymmetrical distribution of proportion estimates across all analyzed results (Fig. S3). Egger’s test indicated significant funnel plot asymmetry, suggesting potential publication bias in the proportion estimates of *Blastocystis* subtype 2, subtype 7, and unknown subtypes (Table S5). The funnel plot demonstrated symmetry in the meta-analysis of *Blastocystis* prevalence estimates from studies using molecular methods alone or combined with other methods (Fig. S4). Egger’s test indicated no significant funnel plot asymmetry (*p* = 0.2466), suggesting no significant publication bias in the meta-analysis.

## Discussion

The estimated prevalence of *Blastocystis* infections among community participants in Thailand was 8.34%. In addition, the subtype analysis revealed that ST3 and ST1 are the most prevalent subtypes in Thailand, accounting for 50.05% and 23.50% of positive samples, respectively. For temporal trends, despite the inclusion of studies spanning several decades, no significant changes in *Blastocystis* prevalence were observed over time.

The high heterogeneity (*I*^2^ = 98.2%) in prevalence estimates reflects differences across study regions, diagnostic methods, and participant types, as shown by meta-regression and subgroup analyses. The findings emphasize that prevalence estimates of *Blastocystis* infections vary significantly across Thailand, ranging from 2.79% in Southern Thailand to 13.54% in Eastern Thailand. This variation may be attributed to differences in environmental conditions, socio-economic factors, and access to clean water and sanitation across regions. The notably low prevalence in Southern Thailand could also reflect the limited number of studies conducted in this region, as well as the use of less sensitive diagnostic methods, such as direct smear alone or combined with formalin-ethyl acetate concentration methods [4, 24, 27, 54, 55]. In contrast, the high prevalence of *Blastocystis* infections in Eastern Thailand may be attributed to the larger number of studies conducted in this area [10, 26, 31, 33, 34, 48, 59, 60, 68, 69, 71, 72, 84], several of which employed culture or molecular methods for detecting *Blastocystis* infections [10, 33, 59, 60, 84].

The subgroup analysis suggested that the prevalence of *Blastocystis* was higher in studies utilizing molecular methods (21.42%) and culture-based methods (22.11%), compared to those relying solely on direct smear (1.79%) or concentration methods (3.43%). Direct smear has the lowest sensitivity for detecting *Blastocystis* in fecal samples, as demonstrated by several studies [11, 14, 15]. Meanwhile, formalin-ethyl acetate concentration methods exhibit similar or slightly better sensitivity than direct smear [14, 15, 26]. For *Blastocystis* detection, culture techniques, such as cultivation in Jones’s medium, appear to have higher sensitivity than direct smear and concentration methods [11, 15]. *In vitro* cultivation of *Blastocystis* has been suggested for detecting *Blastocystis* spp*.* carriage in field studies [34]. Compared with the formol-ether concentration method, *in vitro* culture using Jones’s medium showed a 3.9% positive detection rate, whereas no *Blastocystis* cases were detected using the formol-ether concentration technique [70]. Another study suggested that culture using Boeck and Drbohlav’s Locke-Egg-Serum (LES) medium has the highest sensitivity for detecting *Blastocystis* compared to simple smear or formalin-ether concentration methods [4]. Furthermore, PCR methods demonstrate the highest sensitivity among all diagnostic methods [15, 58]. The sensitivity analysis, which included only cross-sectional studies using molecular methods alone or combined with other methods, estimated the prevalence of *Blastocystis* infections among community participants in Thailand at 22.19%. The prevalence was higher in adults (41.73%) than in children (23.80%). These findings underscore the importance of diagnostic methods in determining prevalence and highlight the potential underestimation of *Blastocystis* infections when less sensitive methods are employed.

For participant-specific trends, the subgroup analysis revealed that the prevalence of *Blastocystis* infections varies significantly among participant groups. Military personnel (29.87%) exhibited the highest prevalence. This finding aligns with subgroup analyses, as most studies in Eastern Thailand involved military personnel [31, 32, 34, 71, 72]. Evidence indicates that these military personnel consumed untreated water, which was associated with a higher risk of *Blastocystis* infections. Sharing food, water, and close-contact activities during training may facilitate transmission [31, 72]. Asymptomatic carriers may contribute to ongoing transmission within military or close-contact settings. Military personnel in close contact with military dogs are a possible risk factor, as some military dogs tested positive for *Blastocystis* infections [32]. Additionally, the association between *Blastocystis* infections and military personnel was linked to the rank of the military personnel. Military personnel with “Private” rank may have a higher risk of *Blastocystis* infections because of higher exposure to drinking tap water compared to other military personnel, such as non-commissioned and commissioned officers, who have options for other sources of drinking water [72].

Besides military personnel, orphans exhibited the second-highest prevalence of *Blastocystis* infections (29.01%). A previous study suggested that the transmission route of *Blastocystis* might primarily involve human feces, as animal feces and drinking water samples tested negative for the organism [46]. Therefore, person-to-person transmission of *Blastocystis* among orphans is likely frequent, potentially due to increased exposure to unsanitary conditions and close-contact living environments. Contributing factors could include poor hygienic practices, inadequate toilet training, playing on unclean playgrounds, and other related activities [49]. Furthermore, childcare workers were also reported to be asymptomatic carriers of *Blastocystis*, potentially transmitting it to orphans [7, 49]. Since both orphans and their childcare workers are often asymptomatic carriers of *Blastocystis*, it is essential to implement institutional cleaning protocols and promote hygiene practices in orphanages. In contrast to military personnel and orphans, subgroup analysis revealed that the prevalence of *Blastocystis* was lower among villagers (6.31%) and schoolchildren (7.50%). Shared living conditions in institutions like barracks or orphanages may increase infection risk compared to villages or schools. However, other factors, such as hygiene practices, environmental exposure, and population characteristics, could also contribute to these differences.

For subtype distribution, the meta-analysis revealed that ST3 and ST1 were the most prevalent subtypes in Thailand, accounting for 50.05% and 23.50% of positive samples, respectively. These findings align with global patterns, where ST3 is often the dominant subtype in human infections [50, 79]. The predominance of *Blastocystis* subtype ST3 in the Thai population may be attributed to several factors. ST3 is known for its strict host specificity to humans, rarely being isolated from other animals, which suggests efficient human-to-human transmission [36]. Additionally, studies have shown that *Blastocystis* colonization is associated with increased diversity and richness of the gut microbiota, which may facilitate its persistence in human hosts [5]. Furthermore, phenotypic variations in ST3 isolates, such as higher growth rates and resistance to harsh conditions, have been observed, potentially enhancing their adaptability and prevalence in human populations [56]. The low prevalence of other subtypes, such as ST4, ST6, and ST7, suggests that these subtypes may have limited transmission or less significance in Thailand’s epidemiological context. Interestingly, mixed and unknown subtypes were also observed, indicating potential genetic diversity within *Blastocystis* populations in Thailand.

The present systematic review and meta-analysis had a few limitations. A major limitation is the extremely high heterogeneity (*I*^2^ > 98%) observed across studies, indicating considerable variation that may stem from differences in geographic regions, diagnostic methods, study populations, and sample sizes. Such high heterogeneity compromises the generalizability of pooled estimates and highlights the challenge of interpreting a single overall prevalence rate across diverse study contexts. Although subgroup and meta-regression analyses identified some contributing factors, substantial unexplained heterogeneity remains. This underscores the need for caution when drawing conclusions from the pooled estimates and suggests that aggregated results may obscure important subgroup-specific patterns. Future research should consider applying more sophisticated analytical approaches, such as stratified meta-analyses or Bayesian hierarchical models, which can more effectively account for complex sources of heterogeneity and provide more context-specific prevalence estimates.

Another key limitation is the presence of publication bias, as indicated by Egger’s test and the asymmetry observed in the funnel plot. This suggests that studies reporting higher prevalence rates may have been more likely to be published, potentially inflating the pooled estimates. The impact of such bias on the overall results should not be underestimated, as it may skew the understanding of the true prevalence of *Blastocystis* infections in the population. While the use of molecular detection methods was associated with reduced publication bias in subgroup analyses, bias remains a concern in studies using other diagnostic approaches. Addressing this issue requires both methodological rigor in future research and increased efforts to publish studies regardless of their findings. Moreover, molecular and culture-based methods consistently yield higher prevalence estimates and should be more widely adopted in epidemiological studies to enhance accuracy and comparability. Finally, targeted public health interventions should prioritize high-risk groups, such as military personnel and orphans, and address regional disparities through tailored strategies informed by local prevalence patterns and diagnostic capabilities.

## Conclusion

This study estimated the prevalence of *Blastocystis* infections among community participants in Thailand at 8.34%, with the highest rates observed in Eastern (13.54%) and Western (10.09%) regions. ST3 and ST1 were the most prevalent subtypes, accounting for 50.05% and 23.50% of cases, respectively. No significant temporal changes in prevalence were detected in the included studies published between 1995 and 2024. To improve diagnostic accuracy, molecular and culture-based methods should be more widely implemented in epidemiological research. Public health efforts should focus on high-risk groups – such as military personnel and orphans – and on reducing regional disparities through context-specific interventions.

## Data Availability

All data relating to the present study are available in this manuscript, Table S1–Table S5 files.

## Supplementary material

*Table S1.* Search strategy for all databases. *Table S2.* Details of included studies. *Table S3.* Risk of bias across included studies. *Table S4.* Meta-regression and subgroup analysis of the prevalence estimate of *Blastocystis* infections in community participants in Thailand. *Table S5.* Egger’s test for funnel plot asymmetry.

The supplementary material of this article is available at https://www.parasite-journal.org/10.1051/parasite/2025042/olm.
