# Supplementary material for: Prevalence and subtype distribution of Blastocystis infections among community participants in Thailand: a systematic review and meta-analysis
Source: Parasite. 2025 Aug 19;32:53. doi: 10.1051/parasite/2025042 (PMC12364436; doi:10.1051/parasite/2025042)
Supplement: Supplementary file 1 — Table S1. Search strategy for all databases. [file parasite-32-53-s1.pdf]

Table S1. Search terms

General keywords

(Blastocystis OR Blastocysti OR blastocystina OR “Blastocystis hominis”) AND (Thailand OR Siam)

PubMed 3 December 2024

No.	Key concept	Search terms	Results
1.	Blastocystis	Blastocystis[Text Word] OR Blastocystis[MeSH Terms] OR Blastocysti[Text Word] OR Blastocysti[MeSH Terms] OR blastocystina[Text Word] OR blastocystina[MeSH Terms] OR “Blastocystis hominis”[Text Word] OR “Blastocystis hominis”[MeSH Terms] OR “Blastocystis hominis”[Text Word]	2,375
2.	Thailand	Thailand[Text Word] OR Thailand[MeSH Terms]	47,521
3.	#1 AND #2	(Blastocystis[Text Word] OR Blastocystis[MeSH Terms] OR Blastocysti[Text Word] OR Blastocysti[MeSH Terms] OR blastocystina[Text Word] OR blastocystina[MeSH Terms] OR “Blastocystis hominis”[Text Word] OR “Blastocystis hominis”[MeSH Terms] OR “Blastocystis hominis”[Text Word]) AND (Thailand[Text Word] OR Thailand[MeSH Terms])	70

Embase 3 December 2024

No.	Key concept	Search terms	Results
1.	Blastocystis	Blastocystis:ti,ab,kw,de OR Blastocystis/exp OR Blastocysti:ti,ab,kw,de OR Blastocysti/exp OR blastocystina:ti,ab,kw,de OR blastocystina/exp OR “Blastocystis hominis”:ti,ab,kw,de OR “Blastocystis hominis”/exp OR “Blastocystis hominis”:ti,ab,kw,de	3239
2.	Thailand	Thailand:ti,ab,kw,de OR Thailand/exp	55,491
3.	#1 AND #2	(Blastocystis:ti,ab,kw,de OR Blastocystis/exp OR Blastocysti:ti,ab,kw,de OR Blastocysti/exp OR blastocystina:ti,ab,kw,de OR blastocystina/exp OR “Blastocystis hominis”:ti,ab,kw,de OR “Blastocystis hominis”/exp OR “Blastocystis hominis”:ti,ab,kw,de) AND (Thailand:ti,ab,kw,de OR Thailand/exp)	82

Scopus 3 December 2024

No.	Key concept	Search terms	Results
1.	Blastocystis	TITLE-ABS-KEY (Blastocystis OR Blastocysti OR	3,328

		blastocystina OR “Blastocystis hominis”)	
2.	Thailand	TITLE-ABS-KEY (Thailand OR Siam)	129,196
3.	1 AND 2	TITLE-ABS-KEY (Blastocystis OR Blastocysti OR Blastocystina OR "Blastocystis hominis") AND (TITLE-ABS-KEY (Thailand OR Siam)	83

Ovid 15 3 December 2024

No.	Key concept	Search terms	Results
1.	Blastocystis AND Thailand	(Blastocystis OR Blastocysti OR blastocystina OR "Blastocystis hominis") AND (Thailand OR Siam) {Including Limited Related Terms}<br>Filter: limit to (ovid full text available and articles with abstracts and original articles)	304

ProQuest 3 December 2024

No.	Key concept	Search terms	Results
1.	Blastocystis AND Thailand	(Blastocystis OR Blastocysti OR blastocystina OR "Blastocystis hominis") AND (Thailand OR Siam)	395

Thai-Journal Citation Index 3 December 2024

No.	Key concept	Search terms	Results
1.	Blastocystis AND Thailand	Blastocystis	13

TCI selection

-Conducted in other countries (n = 5)
-Duplicated with main databases (n = 1)
-Blastocystis in hospitalized patients (n = 2)
-Review (n = 2)
-Full-text unavailable (n = 1)
-Included (n = 2)
