# Supplementary material for: Prevalence and subtype distribution of Blastocystis infections among community participants in Thailand: a systematic review and meta-analysis
Source: Parasite. 2025 Aug 19;32:53. doi: 10.1051/parasite/2025042 (PMC12364436; doi:10.1051/parasite/2025042)
Supplement: Supplementary file 3 — Table S4. Meta-regression and subgroup analysis of the prevalence estimate of Blastocystis infections in community participants in Thailand. [file parasite-32-53-s3.pdf]

Table S4. Meta-regression and subgroup analysis

1. Meta-regression analysis of the prevalence of *Blastocystis* infections among non-hospitalized participants in Thailand

**Covariates**	**tau^2**	**Test for residual heterogeneity, *P* value**	**Residual heterogeneity I^2 (%)**	**Test of moderators, *P* value**	**Number of studies**
Publication years	3.0054	< 0.0001	99.34	0.2758	60
Study design	3.0610	< 0.0001	99.37	0.5835	60
Regions of Thailand	2.1444	< 0.0001	98.88	0.0053	60
Age groups	3.0593	< 0.0001	99.31	0.3035	48
Male percentage	2.8190	< 0.0001	99.13	0.8667	37
Types of participants	2.11	< 0.0001	98.93	0.0900	60
Method for *Blastocystis* detection	1.8630	< 0.0001	98.92	< 0.0001	60

2. Subgroup analysis of the prevalence of *Blastocystis* infections among non-hospitalized participants in Thailand

**Regions of Thailand**	**Province**	**Prevalence estimate (95% CI)**	**I^2 (%)**	**Number of studies**
Overall		8.34 (5.48–12.51)	98.2	60
Eastern Thailand		13.54 (7.41–23.44)	97.9	12
	Chachoengsao	8.93 (4.12–18.29)	88.0	7
	Chonburi	39.25 (35.71–42.90)	79.1	3
	Chanthaburi	9.09 (6.12–13.31)	N/A	1
	Sakaeo	7.78 (6.05–9.95)	N/A	1
	Saraburi	17.49 (13.36–22.56)	N/A	1
Western Thailand		10.09 (2.83–30.17)	98.7	6
	Kanchanaburi	8.90 (0.89–51.47)	98.2	3
	Tak	19.18 (6.27–45.72)	98.6	2
	Ratchaburi	3.35 (2.09–5.32)	N/A	1
Central Thailand		8.85 (4.89–15.48)	96.8	20
	Bangkok	12.71 (7.19–21.47)	95.9	7

	Pathum Thani	14.08 (2.59–50.27)	96.0	4
	Nakhon Pathom	8.89 (5.96–13.07)	93.2	3
	Nakhon Sawan	17.15 (14.31–20.43)	N/A	1
	Ayutthaya	5.91 (3.46–9.91)	N/A	1
	Nonthaburi	10.92 (8.25–14.32)	N/A	1
Northern Thailand		7.11 (1.49–27.91)	98.2	8
	Chiang Rai	51.45 (16.33–85.19)	97.6	3
	Chiang Mai	1.87 (0.79–4.37)	71.8	3
	Nan	1.54 (0.63–3.68)	90.0	2
Northeastern Thailand		4.73 (1.51–13.84)	88.5	6
	Khon Kaen	1.86 (0.24–13.16)	92.1	3
	Nakhon Ratchasima	9.44 (4.75–17.87)	89.7	2
	Loei	12.21 (9.56–15.47)	N/A	1
Southern Thailand		2.79 (1.96–3.97)	39.2	4
	Nakhon Si Thammarat	3.69 (2.47–5.49)	0.0	2
	Songkhla	0.81 (0.20–3.18)	N/A	1
	Satun	2.45 (1.02–5.75)	N/A	1

Note: The results in the table are based solely on single-province studies. Data from multi-province studies are excluded from this table. N/A, not assessed.
