# Supplementary material for: Prevalence and subtype distribution of Blastocystis infections among community participants in Thailand: a systematic review and meta-analysis
Source: Parasite. 2025 Aug 19;32:53. doi: 10.1051/parasite/2025042 (PMC12364436; doi:10.1051/parasite/2025042)
Supplement: Supplementary file 4 — Table S5. Egger’s test for funnel plot asymmetry. [file parasite-32-53-s4.pdf]

Table S5. Egger's test for the proportion estimate of *Blastocystis* subtypes in Thailand

**Subtypes**	***P* value**	**Bias estimate**	**t**	**df**
Subtype 1	0.3241	-1.8655	-1.02	13
Subtype 2	0.0101	-2.4649	-3.1	11
Subtype 3	0.6629	0.6598	0.45	13
Subtype 4	0.5303	0.6306	0.65	12
Subtype 5	Unable to calculate	Unable to calculate	Unable to calculate	
Subtype 6	0.0644	-1.7152	-2.04	12
Subtype 7	< 0.0001	-11.43	-2.4433	13
Subtype 10	0.0908	-0.6867	-1.83	13
Subtype 23				
Subtype 26	0.6395	0.7100	0.48	13
Mixed Subtype	Unable to calculate	Unable to calculate	Unable to calculate	
Unknown subtype	0.0002	-1.6289	-5.25	13
